# Understanding Adoption and Preliminary Effectiveness of a Mobile App for Chronic Pain Management Among US Military Veterans: Pre-Post Mixed Methods Evaluation

**DOI:** 10.2196/33716

**Published:** 2022-01-20

**Authors:** Timothy P Hogan, Bella Etingen, Nicholas McMahon, Felicia R Bixler, Linda Am, Rachel E Wacks, Stephanie L Shimada, Erin D Reilly, Kathleen L Frisbee, Bridget M Smith

**Affiliations:** 1 eHealth Partnered Evaluation Initiative Veterans Affairs Bedford Healthcare System Bedford, MA United States; 2 Center for Healthcare Organization and Implementation Research Veterans Affairs Bedford Healthcare System Bedford, MA United States; 3 Department of Population and Data Sciences University of Texas Southwestern Medical Center Dallas, TX United States; 4 Center of Innovation for Complex Chronic Healthcare Edward Hines Jr. Veterans Affairs Hospital Hines, IL United States; 5 Department of Health Law, Policy, and Management Boston University School of Public Health Boston, MA United States; 6 Division of Health Informatics and Implementation Science Department of Population and Quantitative Health Sciences University of Massachusetts Medical School Worcester, MA United States; 7 Mental Illness Research, Education, and Clinical Center (MIRECC) Veterans Affairs Bedford Healthcare System Bedford, MA United States; 8 Social and Community Reintegration Research (SoCRR) Veterans Affairs Bedford Healthcare System Bedford, MA United States; 9 Office of Connected Care Veterans Health Administration Washington, DC United States; 10 Northwestern University Feinberg School of Medicine Chicago, IL United States

**Keywords:** mobile health applications, pain, veterans, usability

## Abstract

**Background:**

The Veterans Health Administration Pain Coach mobile health app was developed to support veterans with chronic pain.

**Objective:**

Our objective was to evaluate early user experiences with the Pain Coach app and preliminary impacts of app use on pain-related outcomes.

**Methods:**

Following a sequential, explanatory, mixed methods design, we mailed surveys to veterans at 2 time points with an outreach program in between and conducted semistructured interviews with a subsample of survey respondents. We analyzed survey data using descriptive statistics among veterans who completed both surveys and examined differences in key outcomes using paired samples *t* tests. We analyzed semistructured interview data using thematic analysis.

**Results:**

Of 1507 veterans invited and eligible to complete the baseline survey, we received responses from 393 (26.1%). These veterans received our outreach program; 236 (236/393, 60.1%) completed follow-up surveys. We conducted interviews with 10 app users and 10 nonusers. Among survey respondents, 10.2% (24/236) used Pain Coach, and 58% (14/24) reported it was easy to use, though interviews identified various app usability issues. Veterans who used Pain Coach reported greater pain self-efficacy (mean 23.1 vs mean 16.6; *P*=.01) and lower pain interference (mean 34.6 vs mean 31.8; *P*=.03) after (vs before) use. The most frequent reason veterans reported for not using the app was that their health care team had not discussed it with them (96/212, 45.3%).

**Conclusions:**

Our findings suggest that future efforts to increase adoption of Pain Coach and other mobile apps among veterans should include health care team endorsement. Our findings regarding the impact of Pain Coach use on outcomes warrant further study.

## Introduction

Chronic pain is a leading cause of disability [1] and poses a significant, costly problem [2] among US adults, including veterans of the US military. In fact, veterans suffer from pain at disproportionately high rates compared with the general population [3]. Reports suggest that nearly two-thirds of veterans experience pain [3], with even greater pain prevalence observed among certain veteran cohorts [4-6]. This pain experience leads to negative consequences including functional impairment [7], increased stress and mental health concerns [8,9], substance abuse [8,9], risky opioid use [10], impaired sleep [9], decreased quality of life [11], and increased health care utilization [12]. Accordingly, safe and effective strategies are needed to help veterans manage pain.

In recent years, national guidelines for the management of chronic pain have recommended use of nonpharmacological pain management strategies over medications (ie, opioid analgesics) [13], including US Veterans Health Administration (VA) and Department of Defense Clinical Practice Guidelines that suggest frontline treatment options such as behavioral therapy, activity-based therapy, and some nonopioid medications [14]. In line with these guidelines, the VA health care system is committed to identifying non-medication–based pain treatment options and self-management support for veterans who experience chronic pain.

**Figure 1:**
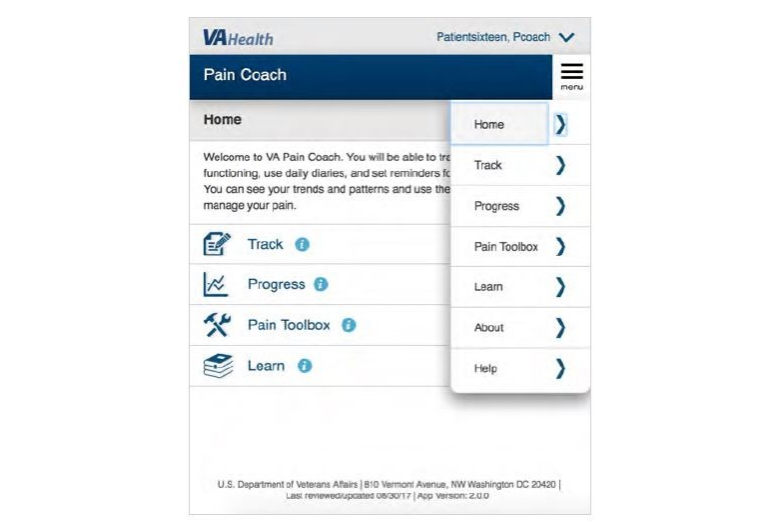
VA Pain Coach interface.

Mobile health apps are a platform for providing accessible self-management tools to patients, and a number of apps are being developed to help individuals manage pain [15-19], including by the VA’s Office of Connected Care (OCC). Recently, the OCC developed the VA Pain Coach mobile health app as a tool to support veterans in managing chronic pain and related care. Through the app, veterans can reference educational resources about pain, including pain management techniques and tools besides medications; track and monitor their pain through a daily pain diary and monthly check-in; and review tables and graphs of changes in their pain level over time [20]. The data that veterans enter into the app are viewable by VA care team members through VA care team–facing apps. A screenshot of the VA Pain Coach app interface, which is a web-based (rather than native) app, is included in Figure 1.

Mobile health apps like VA Pain Coach have great potential for helping veterans communicate with their care team members and self-manage their health. Recent literature suggests that veterans are interested in using mobile health tools [21,22]; however, the adoption of such tools has been limited to date [22,23]. The objective of this evaluation was to evaluate early user experiences with the Pain Coach app and preliminary impacts of app use on pain-related outcomes.

## Methods

### Design

Beginning in summer 2019, we completed a sequential, explanatory, mixed methods, multisite evaluation of the Pain Coach app, wherein we first collected and analyzed quantitative data and subsequently, qualitative data. Specifically, we administered mailed surveys at 2 time points, with an outreach program in between, and then conducted semistructured interviews with a purposive sample of veterans who responded to the surveys.

### Recruitment and Data Collection

We recruited veterans with a chronic pain diagnosis, including veterans who either used or did not use opioid therapy, from 3 geographically dispersed VA Medical Centers located in the Western and Southeastern regions of the United States.

#### Baseline Survey

We mailed the baseline surveys, along with a cover letter and a postage‑paid reply envelope, to veterans beginning in August 2019. We placed follow-up phone calls to veterans who did not return a survey in the mail to maximize their opportunity to participate. A member of our team mailed veterans who completed the survey a US $10 gift card to thank them for their time.

#### Outreach Program

Veterans who completed the baseline survey subsequently received the components of an outreach program about the Pain Coach app. The components included (1) a frequently asked questions (FAQ) sheet about the Pain Coach app that veterans received in the mail, (2) a phone call to veterans from the VA National Telehealth Technology Helpdesk (NTTHD) to offer additional support and troubleshooting with the app as needed, and (3) an informational email sent to the VA care team members of these veterans explaining the Pain Coach app.

#### Follow-up Survey

Beginning in February 2020, we mailed follow-up surveys to veterans who completed a baseline survey and received the outreach program components. Following the methods described in the previous section, we mailed surveys along with a cover letter and postage-paid reply envelope. We again placed follow-up phone calls to veterans who did not return a survey in the mail and compensated respondents with another US $10 gift card.

#### Semistructured Interviews

In accordance with the principles of purposive sampling, we identified a set of criteria (age, gender, geographic location, Pain Coach app use, and use of opioid therapy) with which to target our interview recruitment. We conducted interviews by telephone between April 2020 and June 2020. The interviews lasted 20 minutes to 30 minutes on average and were audio-recorded and transcribed verbatim to facilitate analysis.

### Data Collection Instruments and Measures

#### Baseline Survey

We collected information on veteran sociodemographics including age, gender, race, ethnicity, relationship status, highest level of education completed, living arrangement, and self-reported health status (1=excellent to 5=poor). We measured veteran socioeconomic status by asking veterans to report how hard it is for them (and their family) to pay for basic necessities like food and heating/cooling (1=very hard to 4=not very hard). We also asked veterans to describe the technology that they own (desktop or laptop computer, tablet, cell phone).

Experience with and management of pain variables included prior-week pain intensity measured using a 10-point validated rating scale, ranging from 0 (no pain at all) to 10 (pain as bad as you can imagine) [24]. We measured pain self-efficacy using the Pain Self-Efficacy Questionnaire [25], an established 10-item scale that measures the extent to which individuals are confident that they can perform a range of activities despite their pain. We assessed pain interference with the Patient-Reported Outcomes Measurement Information System 8-item pain interference short form [26], which measures the extent to which individuals felt their pain interfered with their life and activities in the prior week. We also asked veterans to report how much pain had interfered with their sleep in the prior week (1=not at all to 5=very much). Finally, we gathered information on pain outcomes using the Pain Outcomes Questionnaire - Short Form [27], a 19‑item validated scale that assesses pain-related outcomes.

#### Outreach Program

The 1-page FAQ sheet about the Pain Coach app that we mailed to veterans included a summary of the purpose of the app, details about its features, and where veterans could find more information about the app. Veterans also received proactive technical support in the form of telephone calls from the VA’s NTTHD in case they were having difficulty with the Pain Coach app. Callers from the NTTHD followed a script developed by our evaluation team to walk veterans through any challenges they were encountering using the app and troubleshooting to overcome those challenges. The final component of our outreach program was an informational email that briefly summarized the purpose and features of the Pain Coach app and the evaluation we were conducting, which was sent to the VA care team members of the veterans who returned our baseline survey.

#### Follow-up Survey

We asked veterans to report whether they recalled receiving our outreach program components. In addition, we asked veterans to report whether they had used the Pain Coach app. Among veterans who reported having used the app, we assessed self-reported patterns of use.

We also asked self-reported app users about their experiences with and perspectives on the Pain Coach app. Questions asked veterans to report their level of agreement (1=strongly agree to 5=strongly disagree) on factors related to app usability and usefulness and whether the app helped them communicate with their VA care team about their pain. Additionally, we asked veterans to report their level of agreement (1=strongly agree to 5=strongly disagree) on items assessing perceived impact of the app on outcomes (eg, VA Pain Coach helped me to be more engaged in my pain management). Further, we asked veterans to rate their satisfaction with the app (1=very satisfied to 5=not at all satisfied) and their likelihood of recommending the app to other veterans (1=definitely will to 5=definitely will not recommend). Among veterans who reported not having used the app, we asked them to indicate their reasons for nonuse.

We also repeated all questions from the baseline survey regarding veterans’ experiences with and management of pain (please see the Baseline Survey section).

#### Semistructured Interviews

Our semistructured interviews with app users and nonusers followed guides and, reflective of data integration through “building” in mixed methods, included questions intended to elaborate on responses to specific survey items [28]. We asked veterans who reported having used the Pain Coach app about their perceptions of the usability and usefulness of the app, impact of using the app on outcomes and communication with their VA care team, and general perceptions of the app and suggestions for improvement. In our interviews with veterans who reported not having used the app, we asked them to elaborate on their reasons for not having used it.

### Analyses

#### Survey Data

We analyzed survey data using descriptive statistics (means, percentages) among veterans who completed both a first and second survey. We assessed differences in key demographics (eg, age, relationship status, gender) among veterans who reported having used the Pain Coach app versus those who did not use the app using independent samples *t* tests and chi‑square tests. We also examined differences in key outcomes (ie, pain self-efficacy, pain interference, pain intensity, sleep, pain outcomes) on the pre- and post-surveys for Pain Coach app users and nonusers with paired samples *t* tests. Statistical analyses were performed with STATA MP Version 14.2 software (StataCorp, College Station, TX).

#### Interview Data

We analyzed interview transcripts using thematic analysis [29,30]. We first developed a code list based on topics addressed in the interview guide and added emergent codes to the list during the coding process. We also identified exemplary quotes representative of key themes. Two team members analyzed each transcript, first reviewing the transcript independently and then meeting to discuss codes. Any identified discrepancies were resolved during these meetings until complete consensus was reached for all codes. Responses to open-ended survey questions were analyzed using similar procedures.

This work was reviewed by the Institutional Review Boards at the Edward Hines Jr. VA Hospital in Hines, IL and the VA Bedford Healthcare System in Bedford, MA and designated as program evaluation for quality improvement purposes, exempting it from further oversight (VA Handbook 1058.05).

## Results

### Sample

We invited 1668 veterans to participate in the baseline survey; we adjusted this denominator to 1507 to reflect 9 surveys that were returned as undeliverable, 3 veterans who were deceased, and 149 who declined to participate. We received responses from 393 veterans (393/1507, 26.1% response rate). These 393 veterans received our outreach program and were invited to participate in the follow-up survey; we received completed follow-up surveys from 236 veterans (236/393, 60.1% response rate). In the follow-up survey, we asked veterans if they would be willing to complete a semistructured interview to further share their perspectives on the app; 144 (15 app users and 129 nonusers) of the 236 survey respondents expressed willingness to participate in an interview, and we completed interviews with 10 app users and 10 nonusers. In the following sections, we integrate our quantitative and qualitative data, “weaving” and reporting it together according to topics [28].

### Sample Description: Survey Respondents

#### Sociodemographics

Our sample was predominantly male (195/234, 83.3%), White (154/236, 65.3%), of non-Hispanic ethnicity (213/217, 98.2%), married or in a civil union (157/233, 67.4%), and about 64 years old, on average (Table 1). The majority had completed at least some college or vocational school or were college graduates (192/235, 81.7%) and reported being in fair (100/235, 42.6%) or poor (46/235, 19.6%) health.

**Table 1:** Demographic characteristics and technology ownership among survey respondents (n=236).

Variables		Results
**Sociodemographic variables**		
	Age^a^ (years), mean (SD)	63.5 (11.2)
	Age^a^ (years), range	28.0-87.0
	Gender^a^: male, n (%)	195 (83.3)
	Gender^a^: female, n (%)	39 (16.7)
	Race: White, n (%)	154 (65.3)
	Race: Black or African American, n (%)	69 (29.2)
	Race: Asian, n (%)	2 (0.9)
	Race: Native Hawaiian or other Pacific Islander, n (%)	0 (0)
	Race: American Indian or Alaskan Native, n (%)	9 (3.8)
	Race: Other, n (%)	4 (1.7)
	Ethnicity^b^: Hispanic or Latino, n (%)	4 (1.8)
	Ethnicity^b^: not Hispanic or Latino, n (%)	213 (98.2)
	Relationship status^c^: Married or in a civil union, n (%)	157 (67.4)
	Relationship status^c^: Not married or in a civil union^d^, n (%)	76 (32.6)
	Education status^e^: high school graduate or less, n (%)	43 (18.3)
	Education status^e^: at least some college or vocational school (1-4 years), n (%)	175 (74.5)
	Education status^e^: Master’s/professional/doctoral degree, n (%)	17 (7.2)
	Living arrangement: my own apartment or house, n (%)	211 (89.4)
	Living arrangement: friend’s or relative’s apartment or house, n (%)	19 (8.1)
	Living arrangement: other^f^, n (%)	8 (3.4)
	Financial difficulty^c,g^: not very hard, n (%)	110 (47.2)
	Financial difficulty^c,g^: somewhat hard/hard/very hard/do not know, n (%)	123 (52.8)
	Self-reported health status (general)^e^: excellent, n (%)	1 (0.4)
	Self-reported health status (general)^e^: very good, n (%)	17 (7.2)
	Self-reported health status (general)^e^: good, n (%)	71 (30.2)
	Self-reported health status (general)^e^: fair, n (%)	100 (42.6)
	Self-reported health status (general)^e^: poor, n (%)	46 (19.6)
**Technology ownership variables**		
	Does own a desktop or laptop computer^e^, n (%)	184 (78.3)
	Does not own a desktop or laptop computer^e^, n (%)	51 (21.7)
	Does own a table computer (eg, iPad, Kindle Fire)^h^, n (%)	111 (48.1)
	Does not own a table computer (eg, iPad, Kindle Fire)^h^, n (%)	120 (51.9)
	Cell phone ownership^i,j^: smartphone^k^, n (%)	181 (79.0)
	Cell phone ownership^i,j^: non-smartphone cell phone, n (%)	37 (16.2)
	Cell phone ownership^i,j^: none, n (%)	11 (4.8)

^a^n=234.

^b^n=217.

^c^n=233.

^d^Engaged or in a relationship, single, separated, divorced, widowed.

^e^n=235.

^f^School or dormitory, hospital or detox center, nursing home or assisted living, car or street, jail/prison.

^g^How hard is it for you (and your family) to pay for the very basics like food and heating/cooling?

^h^n=231.

^i^n=229.

^j^If you have multiple cell phones, select the one you use most often.

^k^iPhone, Android, Blackberry, Windows Phone, Symbian, or some other type of smartphone.

#### Technology Ownership

The majority of respondents reported owning a smartphone (181/229, 79.0%) or non-smartphone cell phone (37/229, 16.2%) and desktop or laptop computer (184/235, 78.3%); just under one-half (111/231, 48.1%) reported owning a tablet computer.

#### Sample Description: Interview Participants

Veterans who participated in our semistructured interviews were predominantly male (15/20, 75%) and 56.0 years old, on average. Just under one-half (9/20, 45%) used opioid therapy to manage their pain, and one-half (10/20, 50%) reported having used the Pain Coach app.

### Outreach Program

Among respondents, 26.3% (62/236) recalled having received informational materials in the mail about the Pain Coach app, while 51.3% (121/236) did not recall having received these materials (53/236, 22.5% were unsure). About 7.6% (18/236) recalled having spoken with someone by phone from the NTTHD about the Pain Coach app, while 88.1% (208/236) reported that they did not recall speaking with anyone from the help desk (10/236, 4.2% were unsure). Finally, 11.4% (27/236) reported recalling having had communication about the Pain Coach app with their VA care team members in the past few months, while the majority did not recall such communication (193/236, 81.8%) or were unsure (16/236, 6.8%).

### Veteran-Reported Use of the Pain Coach App

Among our respondents, 10.2% (24/236) reported having used the Pain Coach app while most (212/236, 89.8%) indicated not having used the app (Table 2). We compared sociodemographic characteristics among veterans who reported having used the app and those who reported not using it. We did not find significant differences in app adoption due to gender or relationship status among veterans. However, veterans who reported using the Pain Coach app were younger, on average, than those who reported not having used the app (57.6 years vs 64.2 years; *P*=.01).

Our follow-up semistructured interviews to the survey provided an opportunity to explore in more depth the motivations underlying app use. Some interviewees reported turning to the Pain Coach app as a means of tracking their pain or as part of seeking pain management strategies that could complement what they were already doing or could be alternatives to strategies that they had used previously. Others noted that their pain was chronic and ongoing and as such, were willing to try alternative approaches offered within the app to help them manage it. As one male veteran (52 years old) commented:

I definitely have a lot of years with a lot of pain in multiple sites of my body, and I wanted to figure out what, if anything, the app could do to help.

Some veterans also saw the app as a potential alternative to pain medications or taking pain medications throughout the day. As one female veteran (56 years old) explained:

I suffer from migraines. I suffer from back pain, lower back pain, and other pain. I just refuse to take medication because the medication was masking the symptoms. So, I decided if I use Pain Coach, I could have something to kind of sort of guide me along the way. It was a little more user friendly than taking a pill every time you ate.

Use of the Pain Coach app was also associated with changes that veterans were experiencing. One veteran described using the app to make sense of a type of pain he started feeling, while noting that the app might be useful for individuals who are new to managing pain in general. A male veteran (38 years old) noted:

I think that [the VA Pain Coach app] may be beneficial for someone that is dealing with...new pain or has not heard those things before...

Lastly, changes in an individual’s pain management strategy (eg, changes in their medications) also prompted use of the app.

**Table 2:** Veteran-reported use of the Pain Coach app (n=236 survey respondents; n=24 app users).

Pain Coach app use variables		Sample proportion, n (%)
**Self-reported use of the VA Pain Coach app**		
	Yes	24 (10.2)
	No	212 (89.8)
**Which of the following best describes your use of the VA Pain Coach app?^a,b^**		
	I used it once but am no longer using it.	9 (40.9)
	I used it more than once but am no longer using it.	7 (31.8)
	I am still using it.	6 (27.3)
**To the best you can recall, for about how long have you been using the VA Pain Coach app? Or, if you are no longer using the VA Pain Coach app, about how long did you use it for?^a,c^**		
	3 months or longer	6 (26.1)
	Between 1 and 3 months	4 (17.4)
	Less than 1 month	7 (30.4)
	1 week or less	6 (26.1)
**Utilization of VA Pain Coach app tools^a^**		
	Deep breathing	14 (58.3)
	Muscle relaxation	13 (54.2)
	Sleep tips	12 (50.0)
	Manage thoughts	11 (45.8)
	Visualization	10 (41.7)
	Activity pacing	8 (33.3)
	Plan a pleasant activity	7 (29.2)

^a^Among veterans who reported having used the app.

^b^n=22.

^c^n=23.

### Patterns of Use and Usability of the Pain Coach App

Among veterans who reported having used the Pain Coach app, 41% (9/22) used the app once, 32% (7/22) used the app more than once but discontinued use, and 27% (6/22) reported they were still using the app on their follow-up survey (Table 2). One-half or more reported using the app to support deep breathing (14/24, 58%), muscle relaxation (13/24, 54%), and sleep tips (12/24, 50%).

The follow-up semistructured interviews were an opportunity for veterans to provide feedback on features of the app they were using, including the Pain Toolbox, the section of the app that describes different pain management strategies, and the informational resources section of the app. Veterans reported benefits from this content, noting that it helped alleviate the different physical and mental burdens that pain can present, including stress and rumination that can accompany persistent pain. Still other veterans described how the Toolbox and learning resources served as a reminder of pain management techniques that they were previously aware of but had since forgotten:

It reminded me of techniques that I had...used or I had totally forgotten about, so it was good in that aspect.

male veteran, 52 years old

The semistructured interviews also revealed aspects of the Toolbox and learning resources that veterans felt need to be improved. Some expressed concerns about the interface, while others noted that they did not think the content was useful or actionable beyond other health information resources available on the internet. For instance, one male veteran (54 years old) wanted to understand the rationale behind the pain management tools that were included in the Toolbox and why they might be effective at alleviating pain. In his opinion, it was a shortcoming merely to include the instructions or the how-to of the pain management tool without accompanying evidence:

I don’t know the purpose of doing them [the pain management techniques] if someone said, well this is why we want you to count to 10, because we want you to focus on “this,” because obviously there is more to it than just counting to 10.

Related to this point, other veterans felt that the presentation of general pain management strategies in the Toolbox was less effective than if they could provide a description of the pain they were experiencing so that the Toolbox could suggest strategies based on their specific type of pain. As described by one female veteran (60 years old):

Then they got this little tool thing, like, oh, you go to the pain Toolbox. Then, it says activity pacing, deep breathing, manage thoughts, muscle relaxation...But the thing is it doesn’t ask what pain, what was the pain. You can’t use all this stuff.

#### Perceived Usability

More than one-half of veterans who used the Pain Coach app reported that it was easy to use (14/24, 58.3%). One-half (12/24, 50.0%) indicated that they would have liked to have received more information on how to use the app.

Negative feedback about the app’s usability shared during the semistructured interviews stemmed from its web-based platform (ie, the Pain Coach app is not a native app). Log-in issues emerged as a key usability challenge. The lengthy process to go from VA’s Launchpad (the website through which veterans log into the Pain Coach app) to eventually logging into the app itself was cumbersome for some users. One male veteran (aged 54 years) commented:

There are so many steps to even get to where you can log in...even when the system is working, it’s a lot of steps just to log in to get to where you can log something in...You have to launch an app just to get to the app.

Beyond log-in issues, veterans commented on the input-driven nature of the Pain Coach app, including the effort it takes to self-report information about one’s experience of pain. Veterans wanted it to be faster and more efficient to document their pain levels. A male veteran (52 years of age) explained:

I think as far as documenting and everything...it just could be easier, more user friendly, something quick.

### Usefulness, Communication, and Outcomes Associated with Pain Coach App Use

#### Perceived Usefulness

Many veterans found that being able to track their pain levels in the app was useful (11/24, 45.8%). About one-third (8/24, 33.3%) found that being able to set reminders to track their pain in the app was useful and that the app helped them track how their pain was impacting other facets of their life, such as mood and sleep over time. One-quarter (6/24, 25%) of veterans who used the app reported having a better understanding of how to manage their pain because of the Pain Coach app, and 29% (7/24) stated that the app introduced them to new pain management strategies. Just under one-half of veterans (11/24, 46%) found the educational information about how they can manage pain provided in the Pain Coach app to be useful. Approximately one-third (8/24, 33%) stated that they intend to continue using the app in the future, and just under one-half (11/24, 46%) reported being satisfied with the app. More than one-half of veterans reported that they would recommend the app to other veterans (13/24, 54%).

As part of the semistructured interviews, some veterans described feeling that the app and its features fit their needs and noted that Pain Coach can be a resource to facilitate recall of pain management strategies and to help veterans who want to learn more about how to manage their pain. Other veterans, however, felt that the amount of information to manage in the app was too much and at times, was not straightforward. A 38-year-old male veteran explained his experience this way:

I just think it’s a lot of information...too much information. ...you get agitated by trying to find different information and clicking all search feeds.

Other veterans offered specific suggestions to improve the usefulness of the Pain Coach app’s tracking features. Two key themes emerged. First, veterans suggested that it would be helpful to be able to document pain levels multiple times a day as levels fluctuate. One male veteran (54 years old) described using a different health app on his phone, which also did not allow tracking at multiple time points per day and expressed similar concerns about the Pain Coach app:

If I can get into it numerous times a day and track what’s going on, what happened, you know, it would help. But you know, for someone like myself, it’s kind of a pain. Aggravating. Because at different times, I mean all day long, it’s different for me.

Second, other veterans described how being able to track what they were doing while they experienced different levels of pain would be valuable to them. This might include a data entry field where a veteran could note their activities next to their pain ratings. A female veteran aged 60 years described it this way:

First when you...put in the pain [rating]. If they had something right there where you also have to put what activity...what type of activity you [were] doing, like a note.

Veterans also suggested that receiving reminders to use the app and other motivational messages from the app would elevate it beyond being what could feel at times like a data entry tool. As one male veteran (aged 76 years) said:

[If] some kind of little alarm or alert would pop up on the phone, “Hey, take your pain pills.” I mean, not your pain pill, but “Take your, do your app.”

#### Communication

Among Pain Coach app users, few (2/24, 8% strongly agree; 1/24, 4% agree) reported that the app helped them communicate with their VA care team about their pain. Our semistructured interviews with app users corroborated our survey findings about Pain Coach’s influence on veteran communication with their VA care team members. Our analysis revealed 2 main reasons why veterans felt the app did not help with that communication. First, several felt that their VA care team members simply lacked knowledge of the app or did not know that they were using the app. As one male veteran, aged 54 years, questioned:

Do they [VA care team] see the app? I mean, if they see the app, they didn’t tell me because I've seen them since I've logged into the app.

Beyond care team member awareness of the app and its use, other veterans described that the limited impact of the app on communication with their VA care team members was attributable to the quality of communication that already existed. Although some veterans reported good-quality communication with their care team members, others reported difficulties that they did not think the app would address. Veterans recognized that one of the main values of the Pain Coach app was to share information with their VA care team members; however, they questioned the point of using the app if that sharing did not happen. As one male veteran (54 years old) questioned:

Is this something that my primary care physician is going to see and say “Well, we see your pain was up this day.” You know what I mean? Yeah, so—it is nice for me to sit here and log it in, but is it just for me? ‘Cause if it was just for me, I have a pen and a pad and a paper right next to my chair here, so I could just keep track of it myself if I wanted to.

#### Outcomes

Approximately one-fifth (5/24, 21%) of veterans reported that using the Pain Coach app helped them to be more engaged with their pain management and managing their pain was less frustrating for them because of the app (5/24, 21%); 25% (6/24) found that they were able to manage their pain more effectively because of the app.

We compared pre- and post-survey scores on veterans’ self-reported experience of pain (pain self-efficacy, pain interference, pain intensity, sleep, pain outcomes) for Pain Coach app users and nonusers using paired samples *t* tests (see Table 3). Veterans who used the Pain Coach app reported greater pain self-efficacy after using the app (after, mean 23.1 vs before, mean 16.6; *P*=.01); conversely, among app nonusers, self-efficacy scores did not change from the pre (mean 22.9) to post (mean 23.0) periods (*P*=.46). Moreover, while both veterans who used the Pain Coach app (mean 34.6 vs mean 31.8; *P*=.03) and those who did not (mean 30.5 vs mean 28.7; *P*=.001) reported lower pain interference on the post survey as compared with the pre survey, reported pain interference scores decreased more among app users (2.8-point decrease) than nonusers (1.8-point decrease). Of note, the literature indicates that a 2- to 3-point change indicates a minimally important difference on this pain interference measure [31]. In addition, veterans who used the Pain Coach app reported lower (though nonsignificant) pain intensity after using the app (after, mean 7.0 vs before, mean 7.5; *P*=.09); conversely, among app nonusers, pain intensity did not change from the pre (mean 6.6) to post (mean 6.7) periods (*P*=.52). Finally, both veterans who used the Pain Coach app (mean 119.5 vs mean 105.2; *P*=.04) and those who did not use the app (mean 101.3 vs mean 93.3; *P*<.001) reported improvements on the Pain Outcomes Questionnaire - Short Form, although scores among app users improved more than among nonusers.

**Table 3:** Comparisons of veteran outcomes from baseline to post-survey periods (n=236).

Outcome variables	App users (n=24)				App nonusers (n=212)		
	Baseline survey, mean	Follow-up survey, mean	*P* value	Baseline survey, mean		Follow-up survey, mean	*P* value
Pain self-efficacy [25]	16.6	23.1	.01	22.9		23.0	.46
Pain interference^a^ [26]	34.6	31.8	.03	30.5		28.7	.001
Pain intensity^b^ [24]	7.5	7.0	.09	6.6		6.7	.52
Sleep^c^	4.3	4.3	.50	3.9		3.9	.47
Pain outcomes (overall score)^a^ [27]	119.5	105.2	.04	101.3		93.3	<.001

^a^n=212 nonusers.

^b^n=195 nonusers.

^c^n=203 nonusers.

### Reasons for Nonuse

In addition to understanding the experiences of those veterans who used the app during our evaluation period, we also gathered data from veterans who did not use the app to understand reasons for nonuse. The most frequent reason reported by veterans for not using the Pain Coach app was that their health care team had not talked with them about it (96/212, 45.3%; Table 4). This was followed by 22.2% (47/212) of veterans reporting that they did not use the app because they were not aware of it, 16.5% (35/212) did not think it would help them manage their pain, and 16.0% (34/212) reported that they are not comfortable using apps.

**Table 4:** Reported reasons for nonuse of the Pain Coach app (n=212).

Reasons for not using the app	Sample proportion, n (%)
My health care team has not talked with me about using the app	96 (45.3)
Not aware of the app	47 (22.2)
I do not think it would help me manage my pain on my own	35 (16.5)
I am not comfortable using apps	34 (16.0)
I do not see why it would benefit me	28 (13.2)
I do not have a reliable device on which to use the app	26 (12.3)
I have concerns about entering my information in the app	21 (9.9)
I had difficulty accessing the app (eg, downloading, forgot login/password)	19 (9.0)
Not enough information about the app	14 (6.6)
I do not think it would help my health care team to manage my pain.	13 (6.1)
I do not have internet access or stable internet connection	13 (6.1)
Haven't had the time	12 (5.7)
I already have too many apps or receive too much information as it is	11 (5.2)
I do not want to share information about my pain	9 (4.3)
Limited interest in using the app	8 (3.8)
It costs too much to use the app	5 (2.4)
Needs support to use the app	4 (1.9)
Using other pain management strategies	4 (1.9)
I am already using another app to help manage my pain	3 (1.4)

Our semistructured interview data corroborated several of the key findings regarding reasons for nonuse. Limited awareness and having limited information were further described as key reasons for not using the Pain Coach app. In addition, other veterans described having limited interest in and knowledge of how to use the app, citing time pressures, other commitments, and in some cases, a sense of being overwhelmed by other electronic media. As one male veteran (72 years old) commented:

...I deal with too much computer stuff and emails and internet things, and this [is] just one more thing that, you know, I don’t need on my plate and that’s why, you know, I would never really use it.

For this same veteran, like others who reported not using the app, his lack of interest in Pain Coach also tied to his belief that its features and content could not help him manage his pain in lieu of other strategies he already tried:

I’ve been through everything from physical therapy to disc fusion at this point, and so, you know, I’ve done all the different options. If that’s what your coach thing does, it’s kind of like walking through the various options to managing your pain and, you know, tracking it, then, you know, at this point I’ve done everything. [laugh] So, what else is there?

Finally, several veterans who did not use the Pain Coach app explained that having members of their health care team talk with them about the app might have facilitated their willingness to try it.

I: Okay. Great. What else would have been helpful to you in getting started to use VA Pain Coach? P: Probably, my pain management team talking to me more about it.

male veteran, 50 years old

If I was able to talk to somebody, I guess, while I was already at the doctor. If, you know, that time is already set aside, somebody explain to me how it works or what the benefits of it were, it might help me be more willing to use it.

female veteran, 28 years old

## Discussion

### Principal Findings

Increasingly, patient-facing technologies such as mobile health apps are being used to offer pain management strategies to patients [32,33]; however, information about the adoption and impact of such apps among veterans with chronic pain is limited. In this evaluation, we used mixed quantitative and qualitative methods to examine veteran perceptions of and experiences with Pain Coach, a VA mobile health app designed to facilitate pain self-management in the veteran population. Our participants reflect the diverse experiences of veterans managing chronic pain, including those from different geographic regions and those using different pain management approaches (eg, veterans using and not using opioid therapy). Our findings revealed that a fairly “light touch” outreach program yielded limited adoption of this app among veterans and that future mobile health app implementation efforts might benefit from the integration of health care team member encouragement to maximize patient engagement. Still, despite limited use of Pain Coach among the veterans in our sample, we identified important insights regarding usability issues, as well as encouraging, albeit preliminary, impacts of the app on increasing pain self-efficacy and reducing pain interference.

In order to facilitate app adoption among patients, awareness levels of apps must be increased. Our data revealed that traditional outreach approaches including mailed materials and phone calls appear to have limited impact among veterans in this regard. Although the FAQ sheet that we mailed to veterans about the Pain Coach app seemed to reach the greatest number of veterans in our sample, even this component was seemingly limited in its impact. Despite multiple call attempts, the technical support outreach efforts completed by the VA NTTHD did not seem to reach many veterans. Furthermore, although we provided information to VA health care team members about Pain Coach and its functionality via email, veterans reported little communication with their health care team about the app.

Our findings regarding VA care team member and veteran communication about the VA Pain Coach app are particularly important. The predominant reason veterans reported not having used the app was because their care team members had not talked with them about it. This was further corroborated in our interviews with veterans, during which some participants told us that they may have been more likely to try using the app if their VA care team members had recommended it to them. The implications of these findings are significant and underscore the important role that health care team member endorsement and recommendation play in the implementation, adoption, and use of mobile health apps like Pain Coach [34]. These findings further expand on the evidence that endorsement of mobile health apps by health care providers is key to bolstering patient app adoption, including among veterans [22,35].

It is important to recognize that recommending an app to all patients may not resolve all implementation challenges either. As has been discussed in the literature, various considerations need to be made when recommending mobile apps to patients, including a patient’s technical competence, potential privacy issues, and other potential online harms [36-38]. Our data further suggest other characteristics for providers to consider when determining whether a veteran might be a good candidate to use an app like Pain Coach. These include whether the individual is new to the experience and management of pain, whether they experienced some change in their pain or pain management strategies, and whether they might need a reminder of pain management strategies available to them. Similarly, those who have long-standing, chronic pain may be open to additional support to facilitate coping, thus also making them good candidates for app use.

Our evaluation further revealed insights about veteran perceptions of the usability and usefulness of the Pain Coach app. Veterans noted negative perceptions of the web-based platform of the app and in turn, the cumbersome process required to log in (a by-product of the Pain Coach not being a native app). In addition, while some veterans found the informational resources and tracking features in the Pain Coach app to be useful, participants offered suggestions for how these features could be improved, which may be integrated into future versions of Pain Coach or other pain-focused mobile health apps. These suggestions included noting evidence to support the effectiveness of the pain management strategies detailed in the app, facilitating faster and more intuitive pain tracking, allowing users to track their pain throughout the day as opposed to once per day to account for fluctuations in pain, and including a notes field where users could indicate what they were doing when they experienced that level of pain. Veterans also indicated that they would like for the app to send them reminders and motivational messages.

Previous research focused on mobile health apps for pain management also suggests that users value being able to track their pain in the app and want to share this information with their care providers [39]. Similarly, the veterans in our evaluation recognized that one of the main values of the Pain Coach app was to share information with their VA care team members; however, they questioned the point of using the app if that sharing did not happen, and many did not feel the app facilitated communication. These perceptions could potentially be improved if veterans knew that their care team members (1) are aware of the app, (2) know that the veteran is using the app, and (3) know that the veteran is entering data into the app that are available to inform care. These findings are aligned with previous literature noting that patients want to share and discuss the data they enter into mobile health apps with their providers [40,41] and further highlight the importance of provider engagement in patient app adoption and use and subsequent impacts. In order to ensure that mobile health apps such as Pain Coach are able to effectively facilitate patient-care team communication, care team members must be aware that their patients are using such apps.

Finally, and although preliminary, our results suggested that the Pain Coach app may be effective in supporting veteran pain self-management, with users of the app in our sample reporting improved outcomes (ie, increased pain self-efficacy, reduced pain interference) after using the app. These findings are aligned with previous research suggesting that mobile health apps can effectively help individuals manage chronic pain [42] and the use of mobile health apps for pain can facilitate improved pain outcomes [43,44]. This is encouraging and further supports efforts to improve implementation of mobile health apps for pain management.

### Limitations

Our work was limited by lack of randomization and reliance on self-report data, including data on app usage, because reliable app activity log data were not available at the time this evaluation was conducted. In addition, the small number of veterans who reported using the app limits the comparisons that can be made with nonusers and generalizability beyond our sample and underscores the importance of interpreting the results, particularly those regarding impact of the app on veteran outcomes, with caution. In addition, VA Pain Coach is not a native app but rather a mobile web app accessed through an online browser; veterans may have experienced less usability concerns if they had been able to download VA Pain Coach to their personal device(s) rather than having to access a web portal in order to log in. Future work should leverage larger samples of Pain Coach app users, as well as examine the experiences of veterans who tried the app once but did not return, to understand why and how to better support sustained use. Beyond the kind of pre-post design we utilized here, more rigorous evaluations of the effectiveness of the Pain Coach app would require prospective designs with randomization and less reliance on self-report data.

### Conclusions

The results of this evaluation have revealed that traditional outreach approaches (ie, mailed materials, phone calls) had only a marginal influence on awareness of a pain management mobile app among a sample of veterans with chronic pain. Future mobile health app marketing and outreach efforts should be combined with health care team member endorsement, as care team members appear to have a critical role to play in promoting app adoption. Further, addressing the usability issues inherent in apps like VA’s Pain Coach will only serve to advance implementation. Importantly, even though adoption of the Pain Coach app among veterans in this evaluation was quite limited, our results indicate that the app may be useful for self-management of pain (eg, increasing self-efficacy and reducing pain interference), signaling that more rigorous randomized evaluations may be warranted.

## Abbreviations

FAQ: frequently asked questions
NTTHD: National Telehealth Technology Helpdesk
OCC: Office of Connected Care
VA: Veterans Health Administration
